# Baking results in impaired detection of clinically relevant food allergens

**DOI:** 10.3389/falgy.2026.1796342

**Published:** 2026-05-21

**Authors:** Max D. Bermingham, Rhys T. Meredith, Hayley Mills, Sarah Maddocks, Martin D. Chapman, James A. Blaxland, Maria A. Oliver

**Affiliations:** 1 InBio, Cardiff, United Kingdom; 2 The Centre for Health, Immunology, Microbiology and Environment (CHIME), Cardiff School of Sport and Health Sciences, Cardiff Metropolitan University, Cardiff, United Kingdom; 3 InBio, Charlottesville, VA, United States

**Keywords:** allergenicity, ELISA, food allergens, multiplex array for foods, thermal processing

## Abstract

Quantifying food allergens in complex matrices is hindered by thermal processing, particularly in baked products used for oral food challenges (OFCs), where limited data exists on effects on clinically relevant allergens. To address this, an incurred biscuit was prepared, and 12 allergens were quantified using a multiplex-immunoassay array or ELISA. Biscuit dough was incurred with allergenic materials from egg, milk, peanut, almond, cashew, hazelnut, walnut, sesame, and soy at 1,000 ppm total protein. Dough portions (40 g) were baked at 185°C or 210°C for 15 min. Raw dough, whole biscuits, and biscuit middle and edge fractions were extracted using an optimised buffer at 60°C for 15 min. Baking reduced measurable allergen levels compared with raw dough, with the greatest reductions observed for Gal d 1, Gal d 2 (egg) and Bos d 5 (milk) (86%–98%). Ara h 3 (peanut), Jug r 1 (walnut), and Bos d 11 (milk) were least affected, showing 22%–26% reductions. All allergens showed significant intra-biscuit variation, with lower levels at the biscuit edge than the middle. Gal d 1, Gal d 2, and Bos d 5 were most affected, with up to 450-fold higher levels measured in the middle. These findings show heat processing differentially affects allergen quantification within complex matrices. The mechanism of reduction may be attributed to native conformational epitope loss, epitope masking or cleavage, or heat induced loss of solubility, including aggregation, which may impact *in vivo* reactivity. Understanding processing effects may potentially enhance the safety and efficacy of OFC materials.

## 1 Introduction

Thermal processing is known to impact allergen detection (1–5). This is problematic for the detection of undeclared allergens and for foods associated with the treatment and diagnosis of allergy. Baked goods such as biscuits, muffins and cakes are commonly used in oral food challenges and oral immunotherapy (OIT) food ladders, as a vehicle for major allergen delivery (3, 4, 6–12).

Previous studies document that temperatures and conditions associated with baking can result in physiochemical changes in protein structure, including denaturation, polypeptide chain cleavage and amino acid modifications, which can result in the loss or masking of epitopes, and lead to the formation of insoluble proteins and aggregates (13–16). Limited information is known on how specific allergens, the clinically relevant molecular proteins that illicit IgE responses, are impacted by baking. The majority of studies used “total allergen ELISA” kits to demonstrate a decrease in measured allergen content following thermal processing (1, 17–19). The development of allergen-specific detection methods in recent years has allowed for deeper understanding of how clinically relevant allergens are impacted following thermal processing. IgE reactivity and quantification of major milk allergens Bos d 5 and Bos d 11 in muffins used for oral food challenges have been previously investigated. Bos d 5 was highly impacted by baking, whereas Bos d 11 remained more stable and was quantified in mg amounts in baked muffins (3). This poses a differential risk of reaction depending on the allergen to which an individual is sensitised. Moreover, the data revealed that specific allergen content and IgE reactivity varied significantly within a single muffin, between batches, and between recommended recipes for oral food challenges. Similar links between Gal d 1 and Gal d 2 specific allergen quantification, IgE reactivity and clinical presentation when performing egg challenges have been reported (4, 7). Together, these studies highlight the use of immunoassay methods as tools for monitoring allergen reactivity in food preparations to be used for OFC.

Previous studies observed a decrease in measured allergen content following baking, albeit to varying extents (3, 20). The present study used biscuits as an example baked food to compare variation within a baked food sample, and under different baking conditions. The aim was to understand how the detection of specific allergens by immunoassay would be impacted by baking at different temperatures (185°C and 210°C), and how levels would vary depending on sampling location within the biscuit. A model biscuit dough incurred with defined levels of major allergen sources was developed and subjected to two baking conditions. Allergens were extracted using previously optimised methods and analysed using allergen specific multiplex arrays and ELISA (20).

## 2 Materials and methods

### 2.1 Biscuit preparation

A rice-flour-based biscuit dough was prepared that would be analysed raw and baked. Placebo matrices containing no allergen were prepared initially, dually serving as negative control and a base for the preparation of incurred samples. The placebo biscuit dough ingredients and preparation can be found in Supplementary Material S1, Table S01. Biscuit dough was incurred with allergenic source material flour/powders from egg, milk, peanut, soy, cashew, walnut, almond, hazelnut and sesame. The protein content of allergen source materials was determined by Kjeldahl analysis using the standard nitrogen-to-protein conversion factor of 6.25. These materials were then used to spike food matrices to achieve defined levels of total allergenic protein of 1,000 ppm (Supplementary S2, Table S02).

Incurred biscuit dough was chilled to 4°C, 40 g portions were weighed out and rolled into spheres, and stored at 4°C prior to baking at 185°C or 210°C for 15 min in a pre-heated MONO DX deck oven. Baking at 185°C was selected to reflect standard baking for consumption (e.g., lightly golden, firm). These conditions are comparable to baking conditions for biscuit recipes in published oral-food challenges or allergen reintroduction ladders (8, 9, 21). Baking at 210°C for 15 min was selected to reflect over-baking, and deviation from an instructed condition. The resulting baked biscuits measured approximately 1 cm in height and had a diameter of 9 cm.

Following baking, whole biscuits and sub-samples of the edge and centre of biscuits were obtained. A visual representation of sampling is provided in the Supplementary Material S3, Figure S01. Samples were homogenised by combining several biscuits (*n* = 5), or the respective middle/edge of multiple biscuits (*n* = 3), within a single use Ziploc bag and crushing. The resulting crushed biscuits were transferred to an Aigostar 300105KYI coffee grinder and ground on full speed for 30 s. Any material present on the sides or bound in large particles was distributed using a disposable spatula. This process was repeated a further 4 times for a total of 5 mixes.

### 2.2 Allergen quantification by immunoassay

Samples of raw and baked biscuit were extracted in two buffers according to an extraction protocol previously optimised to cover the allergens investigated in biscuit matrices (20). Briefly, samples were extracted in a 1:10 ratio of extraction buffer (e.g., 1 g sample:10 mL buffer), shaking at 175 rpm for 15 min at 60°C in an orbital incubator. Samples were extracted in 0.05 M Sodium Carbonate/Sodium Bicarbonate, 10% Fish gelatin, pH 9.6 for Bos d 11 analysis, and, PBS, 2% Tween-20, 1 M NaCl, 10% Fish gelatin, 1% PVP pH 7.4 for analysis of remaining allergens.

Biscuit samples were quantified for detectable specific allergen content by ELISA for Cor a 9, Gly m 5 and Gal d 1 (available commercially as ELISA 2.0, InBio, Charlottesville, VA). Gal d 2, Ara h 3, Ara h 6, Bos d 5, Bos d 11, Pru du 6. Ana o 3, Jug r 1 and Ses i 1 were quantified using an xMAP based multiplex immunoassay (available commercially as MARIA for Foods custom kit, InBio, Charlottesville, VA), as previously described (20, 22, 23). These immunoassays utilise pairs of allergen-specific monoclonal or polyclonal antibodies in a sandwich immunoassay format, and are calibrated using purified specific allergen proteins.

### 2.3 Statistical analyses

GraphPad Prism Version 10.2.2. was used for all statistical analyses. Statistical tests used are specified in the figure, or table legend where used.

## 3 Results

### 3.1 Impact of baking on allergen detection

**Figure 1:**
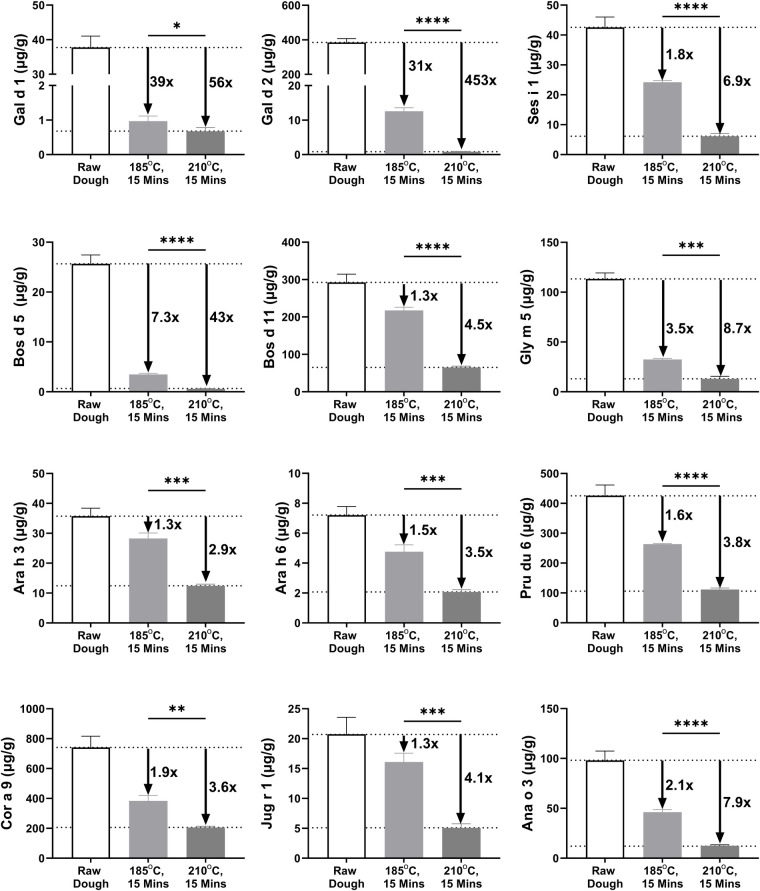
Allergen measurement in raw (unbaked) biscuit dough and baked biscuit. Results plotted as microgram of allergen per gram of sample (µg/g). Fold difference between lowest and highest level of measured allergen plotted to right-hand side. Statistical differences determined by unpaired *t*-test. *P ≤ 0.05, **P ≤ 0.01, ***P ≤ 0.001, ****P ≤ 0.0001). Refer to Supplementary Material, Table S03 for allergen content data.

The impact of baking on allergen recovery was assessed at 185°C and 210°C, baking for 15 min in pre-heated ovens. A comparison of allergen measured in unbaked biscuit dough and biscuits baked at 185°C is presented in Table 1. Full data including both baking conditions are outlined in Figure 1. In each case, percentage or fold reduction is included between biscuit dough and baked biscuit, for each allergen. Further significant (*P* ≤ 0.05) decreases in measurable allergen content were observed in allergens investigated when comparing baking at 185°C and 210°C. The most significant decreases were observed for milk (Bos d 5) and egg (Gal d 1 and Gal d 2) allergens. Detectable levels of Bos d 5 decreased from 7.3-fold when baked at 185 °C, to 43-fold at 210 °C. Conversely, detectable Bos d 11 levels remained high following both baking temperatures, decreasing by 1.3-fold when baked at 185°C, and by 4.5-fold at 210°C. Detectable levels of Gal d 2 decreased >450-fold at 210°C compared to ∼30-fold at 185°C, whereas measurable Gal d 1 decreased by 39-fold at 185°C and 56-fold at 210°C when compared to original unbaked dough. Tree nut, peanut, sesame, and soy allergens also showed marked decreases in measurable allergen levels, and in all cases baking at 210°C resulted in less than half the allergen level compared with samples baked at 185°C.

**Table 1:** Specific allergen content in unbaked biscuit dough and baked biscuits. Results reported as microgram of allergen per gram of sample (µg/g). ^†^Specific tree nut allergens from almond cashew, walnut and hazelnut. Statistical differences determined by unpaired *t-*test.

		Specific allergen content (µg/g)			
Allergenic source	Allergen	Biscuit Dough	Baked Biscuit^‡^	% Reduction	Significance
Egg	Gal d 1	39	0.97	98	****
	Gal d 2	385	11	97	****
Peanut	Ara h 3	36	28	22	*
	Ara h 6	7.2	4.8	33	**
Cow's Milk	Bos d 5	26	3.5	86	****
	Bos d 11	292	218	26	**
Tree nuts^†^	Pru du 6	426	264	38	**
	Ana o 3	98	46	53	***
	Jug r 1	21	16	22	ns
	Cor a 9	741	396	47	**
Sesame	Ses i 1	43	24	43	***
Soy	Gly m 5	113	32	71	****

‡ Biscuits baked at 185°C, for 15 min.

ns, not-significant (*P* > 0.05), *P ≤ 0.05, **P ≤ 0.01, ***P ≤ 0.001, ****P ≤ 0.0001.

### 3.2 Variation of specific allergen levels within baked biscuit

**Figure 2:**
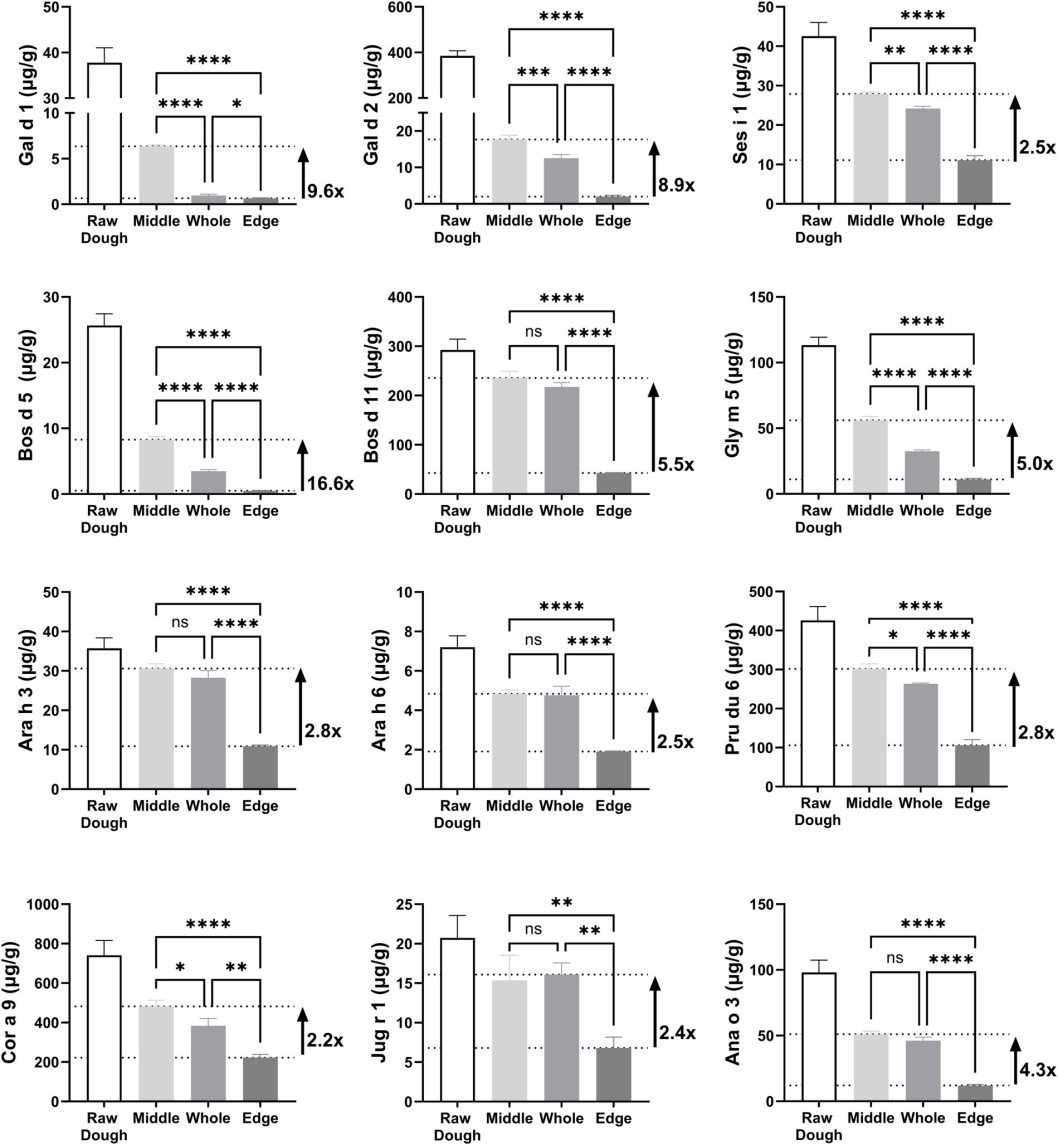
Allergen measurement in raw (unbaked) biscuit dough, whole 185°C baked biscuit, middle and edge. Results plotted at microgram of allergen per gram of sample (µg/g). Fold difference between lowest and highest level of measured allergen plotted to right-hand side. Statistical differences determined by one-way ANOVA. ns, not-significant (*P* > 0.05), *P ≤ 0.05, **P ≤ 0.01, ***P ≤ 0.001, ****P ≤ 0.0001). Refer to Supplementary Material, Table S03 for allergen content data.

Variation within biscuits was assessed through extracting (i) homogenised centres (ii) homogenised whole biscuits and (iii) homogenised edges of biscuits baked at 185°C for 15 min (Figure 2). Results are based on triplicate extractions, with statistical differences determined by one-way ANOVA. Middle-to-edge comparisons were performed for biscuits baked at 210°C, with measured allergen levels documented in Supplementary Material, Table S03.

The results showed significant differences in allergen levels between the middle, and edge of baked biscuits (Figure 2). The greatest differences in detectable allergen levels were observed for animal-derived allergens from egg and milk, varying 8.9-fold for Gal d 2 up to 17-fold for Bos d 5. The greatest variation for a non-animal allergen was observed for Gly m 5 from soy, varying 5-fold. Levels of specific allergens from peanut, Ara h 3 and Ara h 6, were similar, at 2.8 and 2.5-fold respectively. Tree nuts and sesame presented intra-biscuit variations of 2.2 to 4.3-fold. When considering a whole homogenised biscuit vs. the middle, only Ara h 3, Ara h 6, Bos d 11, Ana o 3 and Jug r 1 were found to be comparable (non-significantly different, *P* > 0.05), demonstrating the variability in allergen profile within baked foods, even when homogenised and a representative sample taken.

**Figure 3:**
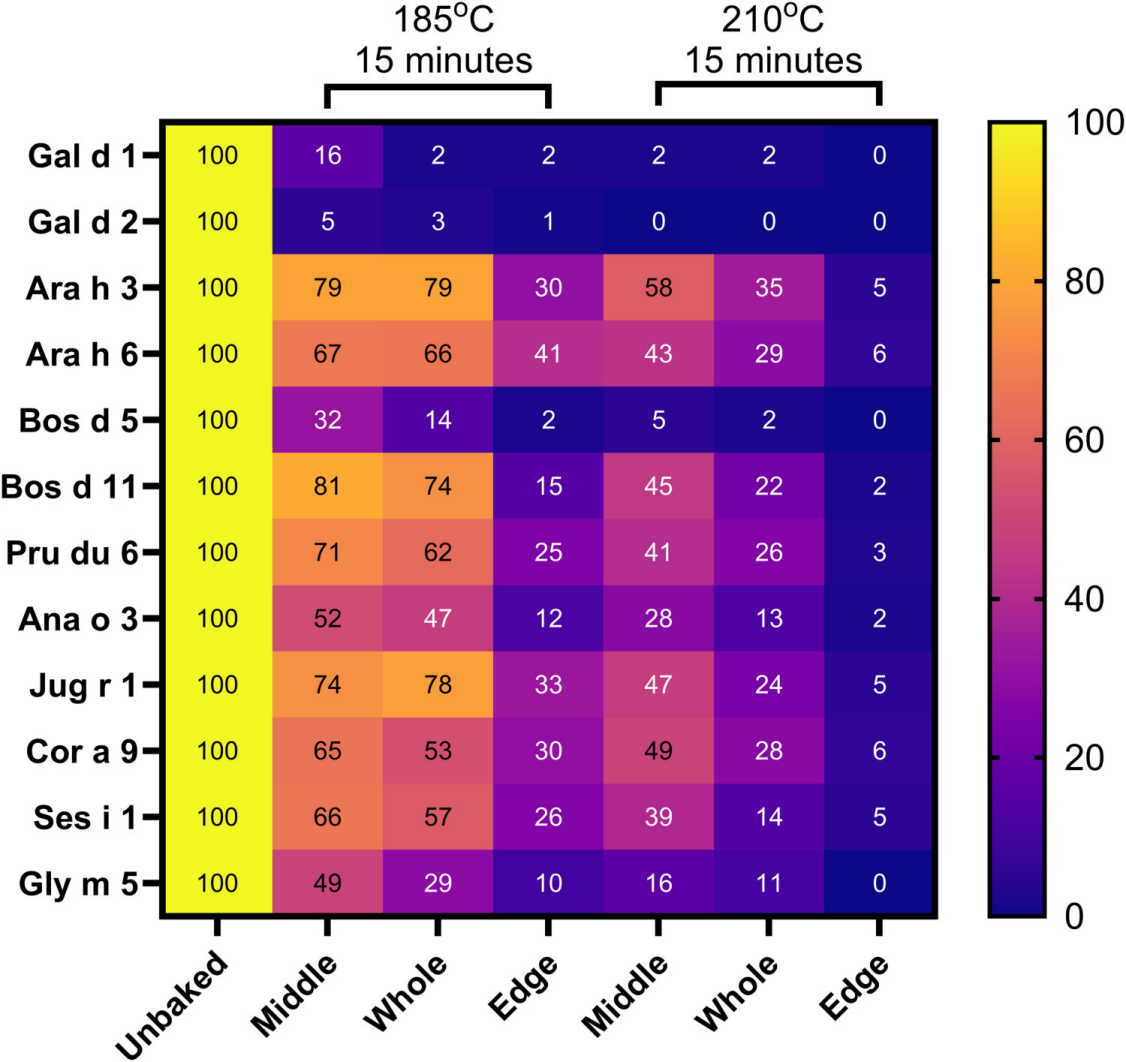
Measured allergen heatmap of percent allergen detected in baked biscuits relative to biscuit dough. Results recorded as percentage recovery relative to unbaked biscuit dough. Refer to Supplementary Material, Table S03 for allergen content data.

Measured allergen from middle, edge and whole biscuits baked at 185°C and 210°C were calculated as a percentage of allergen measured in the uncooked biscuit dough (Figure 3 and Supplementary Material, Table S04). All allergens levels were decreased relative to unbaked biscuit dough following baking, with the largest recovery observed for Bos d 11 at 81% (middle of 185°C biscuit). With exception of Bos d 11, animal-derived allergens from milk and egg presented the greatest susceptibility to heat treatment. Detectable levels of egg allergens Gal d 1 and Gal d 2 were lower in all baked biscuit samples, with recoveries not exceeding 16%. Bos d 5 and Bos d 11 from cow's milk presented varying degrees of stability, whereby levels of Bos d 5 from the whole biscuit decreased to 14%, yet levels of detectable Bos d 11 remained high at 74% relative to unbaked biscuit dough.

Peanut allergens (Ara h 3, Ara h 6), tree nut allergens (Pru du 6, Ana o 3, Jug r 1 and Cor a 9) and sesame allergen (Ses i 1) retained a high level of recovery from the baked biscuit relative to unbaked biscuit dough ranging from 47%–78%, suggesting a higher resistance to thermal processing.

A decrease in measured allergen was also observed from sampling at the edge of baked biscuits, ranging from 1%–41% of the detectable allergen content present in unbaked biscuit dough when considering biscuits baked at 185°C. When in an ‘over-baked, form at 210°C, recoveries diminished to 0%–15%.

## 4 Discussion

The use of baked goods as a vehicle for delivering allergen for allergy diagnosis or treatment is well documented (3, 4, 6, 8–10). Baked matrices are used to mask allergen flavour in “blinded” food challenges (6, 8, 24). The impact of baking is harnessed to deliver allergen doses with presumed lower allergenicity for milk and egg allergens, which can contribute to safer early challenge/therapy stages with lower chance of adverse reaction. Limited studies have investigated the impact of baking on clinically relevant component allergens Gal d 1, Gal d 2 and Bos d 5, Bos d 11 from egg and milk respectively (3, 4). These studies consistently showed that heat processing reduced detectable allergen levels and provided an explanation for tolerance of baked samples by a proportion of food allergic patients. However, some allergens, such as Bos d 11, appear highly heat stable and may explain reactions in individuals sensitised to such allergen, demonstrating the importance of investigation at the component allergen level. Investigations on the effects of baking on the specific, clinically relevant allergens from other allergen sources are limited, yet these are used in baked matrices for oral food challenge (6, 8, 10, 21, 25, 26).

Results from this study demonstrate that detection of clinically relevant food allergens can be significantly impacted upon baking to varying extents. Baking was assessed at two temperatures; 185°C and 210°C to reflect “real life” regular baking conditions, how a product would typically be consumed, and the effect of over/extended baking. Considerable decreases in detectable allergen content were observed for all allergens in 185°C baked biscuits compared to biscuit dough. Extended baking was investigated to cover the hypothetical scenario which could arise because of equipment or human error. It was somewhat surprising that baking at 210°C further reduced allergen levels compared to 185°C. Currently, many OFCs and food ladder samples are prepared by patients or their families. Whilst instructions are provided, it is plausible that variation in baking may occur, whether that be operator (e.g subjective baking by visual judgement, approximations in times or temperature) or equipment (e.g., older, potentially less accurate ovens) related. This calls into question variation that may be observed with home-prepared challenge foods, and whether a place for standardised challenge meals should be pursued, with allergen containing foods processed in a uniform manner.

For milk allergens, the results confirm previous reports that Bos d 11 was resistant to baking and that Bos d 5 was highly susceptible to thermal processing (3). Similarly, egg allergens Gal d 1 and Gal d 2 showed large reductions in allergen levels following baking (31–39-fold) (4). Such results make sense given that Gal d 1, Gal d 2 and Bos d 5 have been demonstrated to denature at temperatures below the baking temperatures studied, and so lead to loss of conformational epitopes or form insoluble aggregates, impacting detection (27, 28). When considering the extended baked sample at 210°C, our data is suggestive that Gal d 1 may have slightly greater resistance to the impacts of heating than Gal d 2, agreeing with previous observations (4). Tree nut, peanut and sesame allergens retained approximately 50% or more detectable allergen content in the 185°C baked biscuits compared to unbaked (∼2-fold reduction), suggesting stability following processing. For tree nut allergens and sesame, these findings are in keeping with literature that suggest a strong level of *in-vitro* reactivity is retained following heat-processing, and may indicate retention of native and readily extractable structure upon baking (29–34). Soy Gly m 5 presented the largest decrease in detectable allergen content for the non-animal allergens, however this is not surprising given previous research has demonstrated major soy proteins Gly m 5 and Gly m 6 denature at ∼72°C to 90°C respectively (35).

The present results showed considerable variation in detectable allergen content when comparing the middle/whole biscuit compared to the biscuit edge, indicating allergens within a given portion of baked food are not uniformly impacted. This finding is as expected given that during baking, foods are heated outside-in, and corroborate observations by Hindley et al. when analysing milk muffins for allergen content (3). Whilst expected, this variability may be relevant when considering baked foods used in the diagnosis or treatment of food allergy, where oral food challenge (OFC) protocols may involve administration of progressively larger portions, beginning with a “small crumb” and followed by fractional doses (e.g., 1/16th, 1/8th, 1/4th of biscuit, muffin or cake) (8, 36, 37). The results suggest that dose levels could vary in measured allergen content depending on where the “crumb”, or respective sample is taken from within the baked food. In the present study, the measured allergen content of a 1 g sample differed by approximately 2.2- to 16.6-fold depending on whether it was taken from the middle or edge of the baked product. The extent to which this analytical variation translates to differences in clinical reactivity is yet to be established, however these findings nevertheless highlight spatial heterogeneity within baked foods as a factor that may warrant further consideration to ensure appropriate incremental dose delivery and uniformity between dosing.

Results of the present study show concordance with Hindley et al. in identifying a reduction in detectable milk allergen content within muffins following baking (3). It is reassuring that the data from these studies agree, however it must be noted these foods have a similar composition and baking condition. It would be beneficial to validate these findings in matrices with a different composition to understand fully whether the reduction in detectable allergen content is a matrix specific result, or universal.

Previous studies on egg and milk suggest a link between allergen detection by IgG based immunoassay, IgE reactivity and clinical presentation. Leeds and colleagues demonstrated that the effect of baking on allergen recovery depends on the food matrix. They reported an egg-allergic individual who tolerated an egg challenge with muffins prepared using the Mount Sinai recipe but later reacted to a maintenance dose when the muffin matrix was modified by substituting banana for wheat flour (7). Subsequent *in vitro* analyses, including quantitative IgG ELISA for Gal d 1 and Gal d 2 and IgE inhibition assays, showed higher levels of Gal d 1, Gal d 2, and IgE binding in banana-containing muffins, indicating increased allergenicity and providing a potential explanation for the clinical reaction (4). It is possible some food matrices may offer “protection” to allergen proteins, adding to stability, or alternatively other matrices may form stronger interactions or enhance processing effects, for example egg and wheat interactions have been observed (38). These results suggest that the different baked food matrices/recipes used for OFC/OIT should be assessed for “available” allergen content as this could have clinical implications.

A potential limitation of the approach used in the present study is whether specific allergens measured by murine IgG-based immunoassay will correlate with IgE reactivity, and whether the epitopes recognised by IgG antibodies are directed against linear or conformational epitopes. Another consideration is whether IgG binding epitopes are lost by denaturation, pyrolytically cleaved or perhaps masked by amino acid modification (e.g., Maillard reaction), or alternatively whether the observed decreases in measured allergen content result from an absence of protein present in solution due to aggregation and precipitation (13–16). Mass spectrometry (MS) could be used to determine whether the target proteins remain in solution and to identify potential linear epitopes. It is plausible that a proportion of the reduction in detectable allergen reflects loss of solubility, as low recovery of allergens from baked matrices in MS has been reported (5, 39). Should MS data identify a lack of protein in solution, one must also consider whether this is a result of poor solubility due to aggregate formation, or due to heat-induced peptide chain hydrolysis of pre-determined peptide target (15). The recent availability of human IgE monoclonal antibodies to specific food allergens, derived from food allergic patients, will allow the effect of baking and other methods of food processing on actual IgE epitopes to be more thoroughly investigated. The human IgE mAb include those directed against peanut (Ara h 1, Ara h 2, Ara h 3, Ara h 6), milk (Bos d 8), egg (Gal d 1, Gal d 2), walnut (Jug r 1) and cashew (Ana o 3) allergens (40). The hIgE mAb to Ara h 2 and Ara h 6 recognise conformational or linear epitopes of known structure which will facilitate future studies (41).

The immunoassay data reported here provide a means to assess and validate recipes and protocols for OFCs and ladder/OIT foods outlined by clinicians or health boards, or commercially produced matrices. Future studies may also wish to study the impact of baking on allergen measurement using a range of matrix bases (e.g., wheat, rice, buckwheat flours). The biscuit base in the present study used rice flour to generate a matrix free of the major 14-allegens. Rice flour was selected as it is specified as an alternative base for use in OFC/OIT preparations in lieu of wheat flour, for patients that must avoid wheat (8). Interestingly, studies have observed an increased OFC failure rate using rice flour as a base instead of wheat, and also lower recovery from wheat-based matrices compared to rice-based (4, 42). These findings are indicative that the wheat-matrix may form stronger interactions with allergens, and have clinical implications. Future studies should look to validate ingredient substitutions in light of this observation, for which IgG based immunoassay methods could be a suitable means.

The biscuit samples analysed in the present study were based on multiple, homogenised samples and so reflect overall trends. Additional studies may wish to investigate this further with repeated sampling within a single biscuit to obtain a complete profile of extremities in measured allergen content. In addition, follow on studies may also wish to investigate whether simulated digestion impacts allergen recovery or impacts IgE reactivity. It is plausible allergen may become “bioavailable” from insoluble complexes when digested *in vivo*, and therefore able to illicit immune response. Allergen ingredients and complex matrices could be subject to the INFOGEST digestion protocol (43) and assessed for quantification by IgG based methods, IgE reactivity and a functional read out (e.g., basophil degranulation) pre and post “digestion”.

## 5 Conclusions

The use of baked goods as foods associated with the treatment and diagnosis of allergy can have significant variations in detectable allergen content depending on sampling location, and through differences in preparation. This could result in varying *in vivo* reactivity to the material, however this should be confirmed with *in vivo* or functional assays (e.g., IgE reactivity, BAT). Measurement of clinically relevant allergen proteins by immunoassay provides a useful tool for assessing allergen distribution and consistency, and can assist in the development of recipes, protocols and commercially produced foods for diagnosis and treatment of allergies.

## Data Availability

The original contributions presented in the study are included in the article/Supplementary Material, further inquiries can be directed to the corresponding author.
