# Supplementary material for: Baking results in impaired detection of clinically relevant food allergens
Source: Front Allergy. 2026 May 21;7:1796342. doi: 10.3389/falgy.2026.1796342 (PMC13233682; doi:10.3389/falgy.2026.1796342)
Supplement: Supplementary file 1 [file Datasheet1.pdf]

Baking results in impaired detection of clinically relevant allergens

Max D Bermingham [1,2],*, Rhys T Meredith [1], Hayley Mills [1], Sarah Maddocks [2], Martin D Chapman [3], James A Blaxland [2], Maria A Oliver [1]

[1]InBio, Cardiff CF23 8HA, UK

[2]Cardiff School of Sport and Health Sciences, Cardiff Metropolitan University, Cardiff CF5 2YB, UK

[3]InBio, Charlottesville, VA 22903, USA* **Correspondence:**
Corresponding Author
mbermingham@inbio.com

S1. Placebo biscuit dough preparation.

Biscuit dough ingredients are outlined in Table S01. Initially, fats, sugars and flavourings were mixed for 10 minutes using a Metcalfe SP-200 Heavy Duty Planetary Mixer set to speed 1. Dry ingredients were subsequently added and mixed for an additional 10 minutes. Material from the sides and bottom of the mixing bowl was manually incorporated and mixed for 10 minutes. Material was transferred to a plastic container and frozen at -20°C prior to the incorporation of allergen.

Table S01. Biscuit matrix ingredients.

Biscuit ingredients	Supplier	Product details	Weight Used (g)
**Gluten free rice flour**	Freee (Doves Farm Foods Ltd)	Gluten free rice flour 1KG Batch JC3201	1931
**Baking Powder**	Asda Stores Ltd	Baking Powder 170g Batch 3177	19
**Vegan butter**	Stork, Upfield Europe BV	Vegan alternative baking spread 500g Batch L33261076	1448
**Caster Sugar**	Silver Spoon, British Sugar plc	Caster Sugar Batch 3170FP	965
**Golden syrup**	Lyle's Golden Syrup, ASR Grp	Lyle Golden Syrup Pouring Batch P3156238	483
**Vanila extract**	Waitrose Ltd	Cooks' Ingredients Madagascan Vanilla Extract 38mL Batch L3242	8

S2. Preparation of incurred biscuit

Source materials covering egg, cow's milk, peanut, soy, cashew, walnut, almond, hazelnut, sesame, mustard, celery, crustacea and fish **can be identified in Table S02.**

Source materials were quantified for total protein content by Kjeldahl analysis Crude (soluble and insoluble) UKAS accredited laboratory. The methods indirectly determine protein content through measurement of nitrogen. Nitrogen content was extrapolated to total protein content by multiplication of a 6.25x conversion factor.

Allergen-free matrices were used as a base to prepare allergen-incurred samples. A 1000ppm high dose was incurred initially, whereby the required amount of each source material required to prepare 1kg of incurred matrix at 1000ppm was calculated (e.g. 3067mg skimmed milk powder with 32.6% protein content = 1000mg protein in 1kg matrix).

Source materials were weighed to prepare a 'source material mix' (SMM). Weighing for the SMM preparation was a critical step in matrix preparation and required the highest level of precision. Materials were weighed using an analytical balance, calibrated with 1mg, 100mg, 1000mg and 200000mg E2 certified calibration weights on the day of weighing. Source materials were weighed to within ±1mg of target value (<0.1%). Source materials were weighed out onto Whatman low static weighing papers and combined in a polypropylene pot. The resulting source material mix was homogenised by vortex mixing at full speed for 2 minutes, placed on a roller mixer for 30 minutes at 60rpm and a final vortex for 2 minutes at full speed.

Table S02. Source material weights for incurring 1000ppm matrices. Total protein (K) determined by nitrogen content, Kjeldahl analysis, 6.25x conversion factor.

***Note**: mustard and salmon source materials included in mix. Specific allergens from these were not analysed in the present study due to assay redevelopment.

Sample	Supplier	Item	Processing notes	µg/g Total Protein (K)
Egg Powder	Sigma-Aldrich	egg powder EO500-1kg	57°C drying	801,000
Milk Powder	Sigma-Aldrich	skimmed milk powder 70166-500g	72°C pasteurisation and drying	326,000
Peanut Flour	Golden Peanut Company	Light roast peanut flour 12% Fat	Light dry roasted, defatted by pressing(1). Light roasting = 121°C for 11 mins, then 157-166°C for 14 mins(2).	543,000
Soy Flour	Sigma-Aldrich	Soybean flour Type 1S9633-500g	'Not roasted, minimal heat treatment'.	516,000
Cashew flour	Beyond the nut	Organic (raw) cashew flour	Raw, ground	212,000
Walnut flour	Hortus Verdi	(Raw) Walnut protein flour	Raw, partially defatted by cold pressing, ground	452,000
Almond flour	Sukrin	Defatted (Raw) Almond flour	Raw, defatted, ground	529,000
Hazelnut flour	Bulgarian Nuts Premium	Hazelnut flour	Blanched, ground	165,000
Sesame powder	Sukrin	Dehusked and defatted (raw) sesame seed flour	Dehusked, defatted, ground	454,000

1000ppm incurred biscuit preparation

Approximately 500g of placebo matrix that had been defrosted and brought to room temperature was added to a stainless-steel mixing bowl, and allergen SMM carefully added on top. Additional placebo matrix was added to bring the total weight in the mixing bowl to 1000g. The matrix was homogenised using a Russell Hobbs GoCreate stand mixer on the lowest speed setting ‘1’. Matrix was mixed for 10 minutes, followed by manual incorporation of material from the side of the bowl and paddle mixer. This was repeated an additional 5 times to give a total of 1 hour mixing.

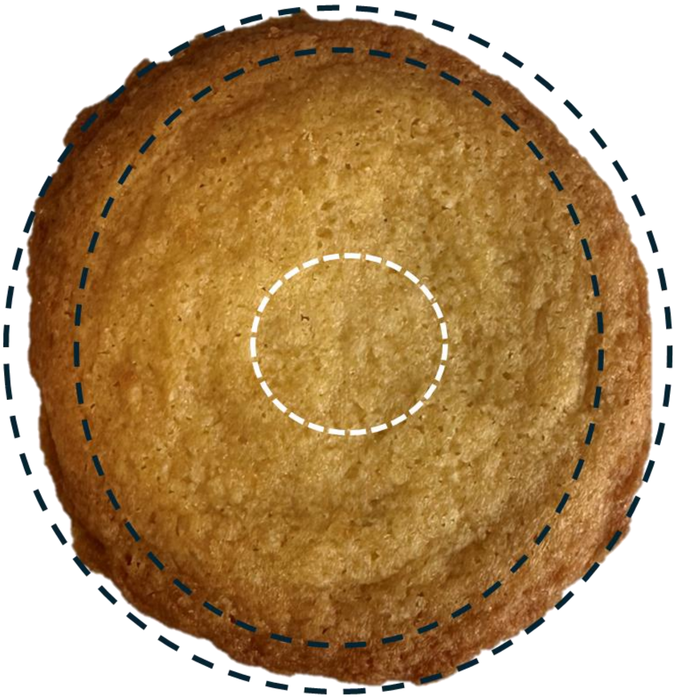

Figure S01. Baked biscuit middle and edge sampling locations. Area within white circle designated middle, and area between blue circles designated edge.

Supplementary Table S03. Allergen content in unbaked and baked biscuit samples. Allergen levels reported as microgram of allergen per gram of food. '<' denotes less than specified limit of detection.

	Allergen content (µg/g)						
		**185°C, 15 minutes**			**210°C, 15 minutes**		
	Unbaked	Middle	Whole	Edge	Middle	Whole	Edge
**Gal d 1**	39	6.4	1.0	0.7	0.9	0.7	<0.313
**Gal d 2**	385	18	13	2.0	0.7	0.8	0.01
**Ara h 3**	36	28	28	11	21	12	1.7
**Ara h 6**	7.2	4.8	4.8	3.0	3.1	2.1	0.4
**Bos d 5**	26	8.3	3.5	0.5	1.3	0.6	<0.01
**Bos d 11**	292	236	218	43	132	65	5.6
**Pru du 6**	426	302	264	106	175	112	14
**Ana o 3**	98	51	46	12	28	12	2.4
**Jug r 1**	21	15	16	6.8	9.7	5.1	1.0
**Cor a 9**	741	482	396	223	367	206	44
**Ses i 1**	43	28	24	11	17	6.2	2.0
**TPM**	1.6	0.6	0.3	0.1	0.3	0.1	<0.01
**Gly m 5**	113	56	33	11	18	13	0.2

References

1. Leonard SA, Ogawa Y, Jedrzejewski PT, Maleki SJ, Chapman MD, Tilles SA, et al. Manufacturing processes of peanut (Arachis hypogaea) allergen powder-dnfp. Front Allergy. 2022;3:1004056. Epub 20221011. doi: 10.3389/falgy.2022.1004056. PubMed PMID: 36304076; PubMed Central PMCID: PMC9592818.

2. Filep S, Block DS, Smith BRE, King EM, Commins S, Kulis M, et al. Specific allergen profiles of peanut foods and diagnostic or therapeutic allergenic products. J Allergy Clin Immunol. 2018;141(2):626-31.e7. Epub 20170712. doi: 10.1016/j.jaci.2017.05.049. PubMed PMID: 28709968.
